# ‘It’s being a part of a grand tradition, a grand counter-culture which involves communities’: A qualitative investigation of autistic community connectedness

**DOI:** 10.1177/13623613221080248

**Published:** 2022-03-23

**Authors:** Monique Botha, Bridget Dibb, David M Frost

**Affiliations:** 1 University of Stirling, UK; 2 University of Surrey, UK; 3 University College London, UK

**Keywords:** autistic community, belongingness, community, identity, political connectedness, qualitative research, social connectedness, stigma, wellbeing

## Abstract

Autistic people report greater comfort socialising and easier communication with each other. Despite autism being stereotypically associated with lack of social motivation, an autistic community has been described briefly in the literature but is not well understood. Autistic community connectedness may play a role in promoting wellbeing for autistic people. This qualitative study involved interviewing autistic individuals (*N* = 20) in-person, via a video-based platform, a text-based platform or over email to investigate autistic community connectedness. Critical grounded theory tools were used to collect and analyse the data. There were three elements of autistic community connectedness: belongingness, social connectedness and political connectedness. Belongingness referred to the sense of similarity that autistic people experienced with each other. Social connectedness referred to specific friendship participants formed with other autistic people. Political connectedness referred to a connectedness to the political or social equality goals of the autistic community. Participants described the benefits of autistic community connectedness as being increased self-esteem, a sense of direction and a sense of community not experienced elsewhere. Lack of connectedness involved ambivalence with an autistic identity and/or feelings of internalised stigma. Experiences of autistic community connectedness may have implications for autistic people’s wellbeing, as well as how they cope with minority stress.

**Lay abstract:**

A sense of being connected to other autistic people has been reported anecdotally. Friendships and connectedness may be important to autistic people and beneficial for their wellbeing. Our research aimed to understand the autistic community by interviewing 20 autistic people about their experiences of being connected to other autistic people. Participants were interviewed in person, over video, using a text-based software to type or over email. Participants detailed three parts of autistic community connectedness: a sense of belonging, social connection with autistic friends and political connectedness. The friendships autistic people had with one another were deemed to be very important to participants because it gave them confidence, provided companionship and made them happy. Some participants did not experience connectedness to the autistic community. These participants also found autism to be less important to their identity and had fewer positive feelings about being autistic. This research is important as it raises awareness that community connectedness is viewed as important to this group. It is possible that community connectedness may help protect the mental health of autistic people when they face stigma or negative life experiences in society.

## Introduction

Individuals exist at the heart of complex ecological systems and communities which may include peers, family or geographical communities (Bronfenbrenner, 1996). While ‘community’ used to be described in a restricted sense to describe only geographic locations, definitions have expanded it more broadly as a shared form of identity, not limited by proximity (Douglas, 2010; Slack, 1998). Thus, a community becomes united through a collective identity and a shared psychological space (Deaux, 1996; Douglas, 2010). A shared psychological space can be described as sharing an identity, values or as an emotional cohesion that unites people (Douglas, 2010). This reflects the cognitive and affective components of community; emotional bonds or ideological solidarity (Frost & Meyer, 2012). There are five elements to a sense of community – membership (feeling of relatedness), influence (the ability to make a difference), reinforcement (needs fulfilment), shared emotional connection (Chavis et al., 1986) and conscious identification (Obst et al., 2002).

A sense of belonging is said to be an innate human need and is associated with higher wellbeing (Allen, 2021; Baumeister & Leary, 1995). Thwarted belonging, in comparison, is associated with a risk of suicide (Van Orden et al., 2010). Minority stress (stigma, discrimination and marginalisation) confers an increased stress burden, and fewer social resources with which to cope (Meyer, 2015). Marginalised minorities, however, might also experience an in-group sense of belonging to similar others which offers positive self-comparison (instead of normative judgement) (Crocker & Major, 1989; Meyer, 2003). For sexual minorities, for example, connectedness to the LGBTQ community weakens the relationship between stigma, depressive symptoms and suicidal behaviour (Kaniuka et al., 2019).

There has been little literature on the experience of autistic community connectedness, and yet it is a term that has been used colloquially for some time to refer to autistic people as a collective population. The roots of the community began in the 1980s, with communication between autistic adults seeking the advancement of autistic rights to education and opportunities (Ward & Meyer, 1999). This was the precursor to The Autism Network International (founded by and for autistic people), which united autistic people into a community primarily over the Internet (Bagatell, 2010; Sinclair, 2005; Ward & Meyer, 1999). Thus, the term ‘autistic community’ has existed for decades, but not particularly as a formalised analytical concept. Autistic community may be undervalued as a phenomenon, due to the stereotypes society hold regarding autism, such as disinterest in friendship, and perceived isolation (Wood & Freeth, 2016). Despite this, there is emerging evidence of a vibrant and vastly understudied autistic community, with a rich history (Kapp, 2020). There have been increasing accounts from scholars (Bagatell, 2010; Ryan Idriss, 2021) and autistic advocates and community members (see Kapp, 2020 for a history of the term and community) on how the community developed, but there is little empirical work which focuses on qualitative *experiences* of autistic community connectedness.

In accounts of friendship that exist, there appears to be a benefit in adult autistic–autistic friendships. In Crompton et al.’s (2019) qualitative study, autistic people reported a unique feeling of belongingness in their friendships with other autistic people. Furthermore, in a study of rapport, autistic-only dyads had increased self-report, and perceived rapport, compared to mixed dyads (Crompton, Sharp, et al., 2020). A quantitative study of autistic children’s friendships found that autistic–autistic friendships were equally as ‘advanced’ as autistic–neurotypical friendships, the only difference being that autistic children in mixed friendships were often assigned subordinate roles (Bauminger et al., 2008). Similarly, research investigating communication between autistic and non-autistic people found that communication happens more smoothly and effectively between homogeneous chains of the same neuro-types including for autistic people (Crompton, Ropar, et al., 2020).

In terms of explicitly studying experiences of autistic community connectedness rather than friendships, there is a case study on an autistic teenager ‘discovering’ the autistic community (Bagatell, 2007), and an ethnographic account of an autistic-led community group in a North America city (Ryan Idriss, 2021). Bagatell (2007) describes how a teenager, on discovering the autistic community, had an initial feeling of relief and started creating friendships. However, the participant ultimately felt torn between two worlds – one world in which it was acceptable to be autistic, and the other in which it was not. Furthermore, Ryan Idriss (2021) describes what they observed in an ethnographic project as an enduring ‘autistic sociality’ of autistic people coming together to build an invisible ‘autistic infrastructure’ of support for one and other that – something which confound deficit-based, theory of mind understandings of autism which construct autistic people as inherently socially disordered. Aside from this, there is limited research which explores the relevance of an autism identity (elsewhere known as an autistic identity) for individual, and collective self-esteem, which found that increased identification appeared to be protective for mental health (Cooper et al., 2017), yet this does not elaborate on community identification or experiences, nor what autistic community connectedness might be. More research is needed beyond these studies as explicit understandings of autistic peoples’ experiences of autistic community connectedness do not yet exist. Given community connectedness’ relationship to mental health and wellbeing in other minority communities (Kaniuka et al., 2019), it might prove a useful concept to understand in relation to autism given the high rate of mental ill-health (Hannon & Taylor, 2013; Lai et al., 2019) and suicide (Cassidy et al., 2014).

This study aimed to provide a qualitative investigation of autistic community connectedness. We aimed to conduct this study without a definition of autistic community connectedness to prioritise the narratives of participants in defining this. To achieve this aim, critical grounded theory (CGT) tools (Hadley, 2019; Kempster & Parry, 2014) were used to collect and analyse qualitative data. Grounded theory is useful where there is little to none current theoretical or empirical data on a subject (Åge, 2011; Charmaz, 2008; Glaser & Strauss, 1967). The study aimed to explore if and how autistic people experience autistic community connectedness.

## Method

### Participants

Purposive theoretical sampling (Corbin & Strauss, 2008) was used to adapt to the heterogeneity of the autistic spectrum – data were analysed throughout collection and guided subsequent recruitment. This approach reached a wide array of autistic individuals with a range of communicative and social needs. Face-to-face and online verbal interviews were offered. Non-speaking, and/or situationally mute autistic people who wanted to take part but would struggle with these formats asked for alternative methods; email and text-based interviews were subsequently offered.

Inclusion criteria stipulated that participants had to be autistic (diagnosed or self-diagnosed), over age 18 years, and proficient in English. Reward for participation was inclusion in a prize draw. Participants were recruited online and locally at University of Surrey. Posters were used for local advertising, and digital posters online through social media. While we did not have a particular threshold in mind for how geographically varied the sample should be, participants were involved globally to prevent *completely* culturally situated understandings of autistic community connectedness. The final sample reflects this with more than one-third of the sample living in either North America, Israel, Europe and South America. People interested in participating emailed the lead researcher and were sent information. A time and interview method was arranged for participants. Prior to interview, participants received the information sheet for a second time before signing a consent form. Overall, twenty participants took part, three using alternative methods to participate. Participant ages ranged from 21 to 62 years (M_age_ = 37.2 and SD_age_ = 13.1). Information on gender, age, race, ethnicity, diagnosis and educational attainment is available in Table 1. The sample was mostly white, had a diverse mix of genders, sexualities and education. Specific data on socioeconomic status were not recorded.

**Table 1.** Participant demographics.

Demographics (*N* = 20)		*n*	%
Gender	Male	9	45
	Female	9	45
	Non-binary	2	10
Ethnicity/race	White British	13	65
	Black British	1	5
	White European	2	10
	White American	1	5
	Mixed-race South American	1	5
	White other (New Zealand)	1	5
	Undisclosed	1	5
Autism diagnosis	Asperger’s syndrome^a^	12	60
	Autistic spectrum condition	2	10
	Pervasive developmental delay (not otherwise specified)	1	5
	No diagnosis	5	25
Sexuality	Heterosexual	13	65
	Bi-sexual/pansexual	5	25
	Undisclosed	2	10
Education	Highschool or below	7	35
	Undergraduate	7	35
	Master’s degree	5	25
	PhD	1	5

a Although Asperger’s syndrome is no longer a diagnosis, we have included it as it is the self-reported diagnosis of participants.

Ethical approval was gained from University of Surrey before recruitment. Confidentiality of participants was protected, and pseudonyms are used.

### Interview procedure

The lead researcher conducted the interviews: face-to-face interviews occurred at the University of Surrey (*N* = 9). Online interviews were conducted over ‘ClickMeeting’ via audio (*N* = 8), text message (*N* = 1) or email (*N* = 2). Oral interviews were audio-recorded for transcription. The duration of the interview varied (32–92 min, (mean = 44.23)), excluding three text-based interviews (mean words = 1741). The lead interviewer adapted their language according to the preference of the interviewee with regards to the use of person-first (person with autism) versus identity first (autistic).

The constant comparative approach was used (Corbin & Strauss, 2008; Kolb, 2012). Here, data were analysed from the first interview onwards and compared to questions being asked, to other data collected, and to the framework being developed. Questions were added to reflect incoming data. Where data were identified contrary to the framework being developed, the framework was adjusted to remain grounded in participants’ accounts. The supervisory team (authors aside from the first author) guided the design of the study as well as specific questions on the interview schedule.

The focus on community connectedness was based on the situated knowledge and position of the first author who is autistic, alongside the theoretical and empirical work on community connectedness in the minority stress literature, and so the original interview schedule was created with these concepts in mind. The original interview schedule (available in Supplementary Appendix A) was as broad as possible to capture wider context and prevent leading despite this insider positionality, and so any questions specifically mentioned community were asked last. Three broad domains were included – diagnosis (how people realised they are autistic), identity and community connectedness. Example questions across around diagnosis and process included, for example, ‘did being diagnosed (or suspecting) you were autistic/on the autism spectrum change the way you thought about yourself or your life?’. Questions around identity included ‘do you feel being autistic/on the autism spectrum autism is a core part of your identity? Why so’. Questions about community included ‘do you have other friends that are on the autism spectrum/autistic? What are these friendships like?’

The main topics that were not originally included, but became prominent during the data collection were stigma, stereotypes, representations of autism and ‘sensing’ other autistic people. Questions on these topics were added as they appeared across multiple interviews including ‘How do you think society feels about autism?’, ‘do you think there are stereotypes attached to autism? Why? What are they?’ and ‘do you feel you notice when someone else is on the spectrum/autistic?’. Stigma and identity were exceptionally prominent in the data and to cover these areas in as much depth as possible are discussed elsewhere (Botha et al., 2020).

### Community involvement

The study was led by the first author who is an autistic researcher, under the guidance of a team of non-autistic researchers, and focused on social issues which is of high priority in the autistic community (Pellicano et al., 2014).

### Data analysis

CGT tools were used in this study (Hadley, 2019; Hoddy, 2018). CGT applies grounded theory tools (Charmaz, 2008; Corbin & Strauss, 2008; Glaser & Strauss, 1967) within a critical realist framework (Hadley, 2019). Critical realism posits an ultimate reality which exists independently of interaction with it, but also that all descriptions of reality are mediated through meaning and context (Bhaskar, 1997). Using CGT, researchers relate experiences between participants, but they also abstract their accounts into the contextualisation of the ecological systems the participants may or may not be aware of (Hoddy, 2018; Kempster & Parry, 2011). For a further explanation of CGT methods in the study, please see Botha et al. (2020). In line with grounded theory tools, the literature review was conducted after data analysis was complete to allow for the prioritisation of participant narrative over the prior knowledge of the researchers involved. This does not mean that the researchers were blank slates to the topic (we all acknowledge our prior knowledge by virtue of our positions), but rather that we did not want to shape the data according to prior theory, and instead build theory based on the data from these interviews instead.

We used NVivo 10 and 11 (QSR International, 2015) to manage and analyse data. The first author coded the data, allowing for continuity between interviewing, coding and initial ideas of how the data related to other participants. All data from all interviews were coded entirely to ensure participants or narratives were not favoured. The coding, interpretation and write-up of the results were subsequently discussed by the supervisory team, who met frequently to identify any potential differing interpretations of the data. As such, the wider team guided the project throughout.

Grounded theory coding techniques were used – open, axial and selective coding (Hoddy, 2018; Kempster & Parry, 2011). Open coding consisted of coding the data line-by-line according to people’s own understandings of phenomena (Hoddy, 2018) and positing links. Axial coding started after interviewing three participants, and included highlighting regularities between data (Charmaz, 2006; Corbin & Strauss, 2008; Hoddy, 2018). During axial coding, we made explicit the relationship between open code categories describing possible mechanisms. During selective coding, core categories were highlighted (Hoddy, 2018; Vollstedt & Rezat, 2019) and their relationship to other categories were made explicit. The relationships between codes and categories were based on the commonalities and contrasts between participants and how they narrated their experiences. Some relationships were based on overt and direct data where multiple participants posited relationships between phenomena themselves, for example, between salience of identity and closeness to the autistic community. Whereas other relationships were based on the key difference in how the participant describe themselves or their experiences, in comparison with the wider data set; for example, the relationship between internalised stigma and autistic community connectedness. The final stage involved abstracting these data upwards relating them to other systems in people’s lives (Hoddy, 2018). The whole process of coding was iterative, and involved moving forwards and backwards between stages to generate a cohesive model as more data were collected, and as the schedule and focus of participants shifted, making the whole process highly adaptive participants narratives. The final model being a product of iterations means that it closely reflected the overall data. This model was then compared to existing literature in a final stage of abstraction to see the ways in which these data contrasted to wider community literature.

### Reflective journaling

Reflective journaling (Janesick, 2015) was used for retrospection, to ensure the coder’s epistemic responsibilities were met and for transparency. Epistemic responsibility refers to a process of active acknowledgement and recognition of the impact and implications of ones’ interpretations and work (Barad, 2007). Given that critical realism acknowledges that phenomena are shaped by perspective (Bhaskar, 1997), this openness and transparency is critical.

## Results

**Figure 1.**
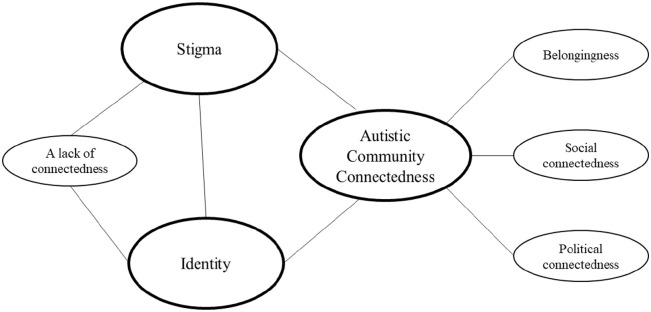
Overview of results. Core categories are bolded ovals, categories are in non-bold ovals, while the relationships between categories are described with lines.

Core categories identified were autistic community connectedness, identity and stigma. An overview is presented in Figure 1. While the research was designed to understand autistic community connectedness, participants’ engagement with the concept evolved over the course of the interview. The discussions of community emerged organically in the initial generic part of the interview and were further refined in the later part of the interview in response to direct questions about community. Our analysis indicated that autistic community connectedness consisted of belongingness, social connectedness and political connectedness – three subdomains of an overarching concept of autistic community connectedness. Belongingness related to an emotive feeling of recognising similar others. Social connectedness reflected the social capital provided by social connection to autistic people. Political connectedness was a goal-orientated connectedness whereby participants focused on rights acquisitions and advocacy. Being connected in one domain did not mean being connected in all. There were benefits attached to being connected to the autistic community such as easing loneliness, improving confidence and generating social change.

Identity and stigma were core categories because they related to connectedness (or lack thereof). Stigma in non-autistic communities pushed autistic individuals to autistic community connectedness, while ambivalence towards autistic identity and internalised stigma seemed to relate to a disconnect; thus, this relationship appears reciprocal. Some aspects of autistic community connectedness would exist regardless of stigma and marginalisation, but political connectedness exists *because* of it. Thus, stigma relates to autistic community connectedness (potentially pushing people towards it), while autistic community connectedness also relates to stigma, by potentially disrupting stigma. Similarly, importance of autistic identity seemed to relate to autistic community connectedness, while a lack of autistic identity related to a lack of connectedness. It should be noted that while identity and stigma are core categories, below they are integrated and discussed throughout because they describe the relationship between other subcategories of data and were pervasive. For an in-depth exploration of this stigma and identity data, see Botha et al. (2020).

### Autistic community connectedness

#### Belongingness

Belongingness appeared to involve feelings of similarity, a sense of a ‘tribe’^1^ among autistic people (reflecting language from the book NeuroTribes (Silberman, 2015)). Participants described feeling connected to and accepted by other people who are autistic or neurodivergent. The similarity was expressed in terms of being on the same wavelength and instant connection: ‘There is an instant connection, I feel like we’re kind of the same . . . (my autistic friend) talks about things the way I think’ (Andrew, 22, Black British, male, diagnosed).

Participants described feeling accepted for their ‘quirks’ not despite them. Participants discussed a constant rejection until discovering an autistic community:

I could relate to everyone on there really quickly, which was odd for me . . . There is just a feeling, I can recognize myself in other [autistic] people . . . (Ava, 35, Peruvian American, female, seeking diagnosis)

Participants described being able to sense similar others. Most could not identify what it was that made them sense other autistic people, describing it only as a gut feeling, or ‘just a sense’, but other participants could:

I can sense [other autistic people], there is something just off, not in a bad way, about their timing. But yeah just like, like something about their timing . . . I have off timing, too. It’s not like a great name but you know in the way of continuing a conversation or something, I mean like talking for a really long time. Its also a feeling, I don’t know, I feel I can recognize myself in autistic people. (Ava, 35, Peruvian American, female, seeking diagnosis)

Importantly, participants described gravitating towards people who are different and ‘weird like them’ describing these connections as instant and valuable:

I sense that we have a lot in common. In those cases, I am very attracted to them. It already happened that after a minute’s conversation I already felt closer to such a person than to people I had known for decades. (Abi, 47, Isreali, male, diagnosed)

Similarly, connecting with other autistic individuals was routinely described as being easier or providing more success than with non-autistic individuals:

I’d say it is easier to connect to people on the [autism] spectrum . . . I have a better success rate. (Ava, 35, Peruvian American, female, seeking diagnosis)

#### Social connectedness

Social connectedness was the specific social connections formed with other autistic people. While belongingness was a general feeling that the autistic community was ‘home’, friendship constituted the individual relationships created in these spaces and referred to *specific* people rather than the community in *general*. Participants discussed how accessibility of spaces which cater to autistic people’s needs facilitated social connectedness. Giving and receiving advice was a part of social connectedness.

Participants had appreciation for all friendships but felt there was something unique to autistic–autistic or autistic–neurodivergent friendships. For example, one participant argued that neurotypical people can be good friends, but do not understand:

Neurotypical friends, whilst they can be good, they just don’t get it. (Carley, 21, female, white British, diagnosed)

Participants described the rapid expansion of forums for autistic people, which demonstrates the demand for spaces:

I joined this particular forum, I joined when I was about one in 10 or 20 . . . And over . . . three or four months, I’ve seen it grow to . . . about 600 people. (Emma, 40, white, female, seeking diagnosis)

The predominant ‘space’ where social connectedness occurred was the Internet, which facilitated connections regardless of geography, and removed constraints of social community (eye contact, body language and speaking). Accessibility was frequently mentioned as environments are rarely designed with neurodivergent people in mind, making them overwhelming, confusing or stressful. Community events (offline) were also mentioned though. Participants looked for autistic-run events:

[I am] participating in events which are organised by autistics, for autistics – meetings, hikes, Autistic Pride Day events, Autscape, Autreat, etc. (Abi, 47, Isreali, male, diagnosed)

I run a group for autistic adults each month, and we see each other at the meetings. (Polly, 32, white, female, diagnosed)

Part of social connectedness was giving and receiving advice, allowing the opportunity to learn about oneself. Topics of advice included handling experiences with neurotypical people, sensory overload and raising autistic children:

I am a part of communities . . . to learn how to deal with neurotypical people and to offer advice and support to parents of people like us. (Maximillian, 54, male, white British, suspected)

There was considerable variance in the friendship experiences described by participants. Most described friendships with neurotypical and autistic people, others only with other autistic people or neurodivergent people. Some who described only having neurodivergent or autistic friendships questioned how any autistic person manages to have neurotypical friends, indicating sense of incompatibility:

I think . . . how do they do it? How do they have neurotypical friends? (Charlie, 29, gender non-binary, white British, diagnosed)

Participants described an appreciation that friendship looks different between autistic people, but were highly appreciated:

I’ve made some friends there [at Autscape] . . . we rarely see each other outside of scheduled meetings . . . sometimes we will talk over the internet . . . it’s probably not quite how other people would see a friendship but I’d get very overwhelmed if they wanted to talk all the time (May, 35, white British, female, diagnosed)

#### Political connectedness

Political community connectedness was a goal-oriented subdomain of autistic community connectedness. Participants described varying levels of political connectedness, meaning some were engaged highly with belongingness and social connectedness, but did not express a high political connectedness, while fewer others, took political steps (such as signing petitions) without expressing a high belongingness or social connectedness to other autistic people. This suggests belongingness, social connectedness and political connectedness individual subdomains of a larger overarching construct rather than a single construct. Similarly, one participant summed up the difference between personal and political engagement in saying that some autistic people with whom he shares interests are his friends, while those whom he shares goals with are comrades.

Rights acquisition was a part of political connectedness. It describes the political movement in which autistic individuals come together to access equal rights. The goals are, about gaining rights, such as a ban on fake ‘cures’, ensuring all autistic children are in appropriate education, and that access to diagnosis and/or support is a global right:

There’s been a rise on MMS [bleach] treatments on autistic children. I recently signed [a petition] banning them. (May, 35, white British, female, diagnosed)

the fact there’s been quite a strong push towards, you know, women finding their diagnosis, because we’ve been kind of overlooked in the past quite a lot. (Emma, 40, white, female, seeking diagnosis)

Political connectedness also involved wanting to be seen as equal, fighting to end stigmatising campaigns, and against the desire to normalise autistic people. Participants described writing to politicians to protest events, like the roll back on autism diagnoses. This fits with the anti-austerity narrative in grey literature with autistic people fighting against the roll back of disability services in the United Kingdom (Pring, 2019).

Furthermore, participants discussed making decisions about what research they would take part in based on political agendas around autism – disagreeing with the concept of curing autism:

I avoid cure research because . . . I feel that all such research hinders the acceptance of autistics as a social minority group. (Abi, 47, Isreali, male, diagnosed)

Concerns about, and deliberate attempts to avoid any genetic research:

It depends on the research and who does it as well, because the medical side of it, I’m kind of wary because there’s a lot of focus on people trying to find the cause of autism, because you know they found the cause of Down Syndrome and look what happened. Like what if there is a prenatal test for autism? What’s going to happen to autistic people? So I kind of shy away from genetic research and the like. The idea we shouldn’t exist is awful. (Charlie, 29, gender non-binary, white British, diagnosed)

The motivations seemed to be about participating in ‘good research’ which helps other autistic individuals:

If it’s a topic that I think is important then I’d be more likely to participate . . . also if the researcher is autistic . . . autistic researchers are more likely to know what’s actually relevant. But people research cures and how to make autistic people more acceptable to non-autistic people. Reasons for a cure are never anything to do with us. (May, 35, white British, female, diagnosed)

Participants described how neurodiversity can forge connections between other minorities to advance the goals of everyone, showing a political motivation spanning multiple disadvantaged social identities:

I view everything from a neurodiversity perspective, and therefore I do my best to cooperate with various social minorities, to promote solidarity . . . and to advance the rights of all of us. [I have] a desire to join other autistics in discussions about how to promote awareness and acceptance of ourselves, as a social minority group, within larger society. (Abi, 47, Israeli, male, diagnosed)

As specific examples, participants discussed the intersection of race and gender, and autism:

Whenever they show a person of colour they’re shown as more classically functioning . . . less verbal and less able. (Charlie, gender non-binary, white British, diagnosed)

As well as culturally situated stigma:

In Nigeria, mental illness and [disability] are downplayed . . . you know, just pretend to be normal. (Andrew, 22, Black British, male, diagnosed)

Some described political autistic community connectedness as being connected to a power grid of activists working to promote social justice, while others embraced it as a grand counter-culture. These descriptions hold power and pride in resistance:

I’m a part of that wider tradition of disability rights activism, basically with what I do it like through the autism stuff, but then add to it, neurodiversity . . . It’s being a part of a grand tradition, a grand counter-culture which involves communities that have been in some cases oppressed by willing people. (Luke, 23, white, British, Male, diagnosed)

#### Benefits of community connectedness

Benefits of autistic community were many and varied, according to the type of connection. Although the subdomains were distinct, taken together, these subdomains gave purpose and joy to autistic people, and ended a social isolation they experienced in neurotypical communities:

I was very isolated and then I met autistic people. (May, 35, white British, female, diagnosed)

The benefits of belongingness and social connectedness included learning about oneself, offering and receiving advice, making connections and friends, and having a ‘home’. The space among autistic people was presented as safe, validating and supportive:

I think, knowledge of where am I and the [community] are so helping me understand myself more. And it’s helping me be more forgiving of my weaknesses. (Emma, 40, white, female, seeking diagnosis)

Political connectedness gave individuals a sense of purpose and a feeling of control; furthermore, it gave them a network of individuals who were fighting for similar goals:

It gave me a social network and a cause to work towards . . . It gives me a sense of belonging and of fulfilment because I feel I am contributing towards improving the quality of the lives of many fellow autistics. (Abi, 47, Isreali, male, diagnosed)

#### A lack of connectedness

Three participants experienced disconnectedness from the autistic community, with one hoping to become connected with time. Factor seemed to be that autism was not central to their identity, internalised stigma and/or a turbulent diagnosis process with disparate parental responses.

#### Lack of centrality of autistic identity

Not holding a diagnosis closely in terms of personal identity appeared to be important to a lack of connectedness. A diagnosis later in life appeared important to this disconnect:

If I stop and reflect, I have to remind myself of [being autistic] sometimes . . . It’s not part of my core identity . . . Maybe if I were diagnosed younger it would be. (Michael, 55, white British, male, diagnosed)

#### A turbulent parental response to diagnosis

A turbulent parental response to diagnosis was also important – two participants who had been diagnosed as children described themselves as autistic but struggled with the identity as autistic due to different responses from parents. In both cases, mothers had been supportive during, and post-diagnosis but fathers had been upset or angered about the diagnosis:

Both my parents gave really different approaches. My mum was very much on it . . . she literally did all the research. My dad, he’s more laid back and part of Nigerian culture . . . That’s how my dad approached it with me . . . I was also good at math, so he less likely believed there was something actually wrong with me . . . he kept pressuring me, just be normal, be normal, there’s nothing to it, be normal. (Andrew, 22, Black, British, male, diagnosed)

Interestingly, both participants questioned whether they really were autistic, asking whether they were ‘too high-functioning’. Both talked about becoming more interested in the autistic community but worried they would be considered ‘imposters’. These participants talked about connections to neurotypical communities, but also described themselves as not really belonging in them either.

#### Internalised stigma

Among participants on the outskirts of the community or unconnected, one of the biggest concerns about accessing community spaces was fear that the other individuals there would be ‘more autistic’. The language they used might also suggest internalised stigma because they reflect the endorsement of negative anti-autistic stereotypes, as well as a psychological distancing of the self from other autistic people:

I have Aspergers and I’m extremely intelligent and joined MENSA but I’m not like other autistic people . . . Also there is a worry that what if they are not high functioning people and I couldn’t relate to that so. I mean, the other people with autism, smell is a big problem because a lot of them aren’t very hygienic . . . I couldn’t stand to be near them at all. (Michael, 55, white British, male, diagnosed)

Furthermore, this quote also pointed towards the concept of ‘Aspie Supremacy’; the idea of the superiority of a certain kind of autistic person over *other* autistic people based on normative ideas of intelligence, economic ‘productiveness’ and perceived utility to society (de Hooge, 2019) – something which has pervaded autism since Hans Asperger’s categorised autistic people by their perceived utility and burdensomeness deeming only some autistic people as worthy of life or personhood (Czech, 2018; de Hooge, 2019).

#### Disconnectedness from both communities

One participant appeared completely disconnected from both neurotypical communities and autistic communities, feeling like he did not fit into neurotypical communities and was always considered ‘weird’, but simultaneously, did not feel there would be benefits to connecting with the autistic community:

I don’t really have a family any longer. I don’t have any friends. No-one to tell really and no-one cares . . . I once joined up with something called Wrongplanet, but because of the way I am, I didn’t really see the point in trying to talk to other people. (Allen, 35, male, New Zealand, diagnosed)

## Discussion

This study aimed to develop a construct and understanding of autistic community connectedness using grounded theory tools. Our analysis of participants’ lived experiences indicated that autistic community connectedness consisted of three different subdomains of community – belongingness, social connectedness and political connectedness. Each domain provided something unique to the overarching construct of autistic community connectedness in participants’ lives. Belongingness related to a feeling of seeing oneself in someone else. Social connectedness consisted of the specific friendships and social capital. Finally, political connectedness was a goal-orientated domain of acquiring rights and provision for themselves and other autistic people. Disconnectedness was apparent in a few participants and related to either a lack of salience in autistic identity, ambivalence with the autism diagnosis, or potentially internalised stigma about how *other* autistic people might be. Tentatively, it appears that while internalised stigma acted as a barrier to autistic community connectedness, perceived external stigma pushed autistic people towards autistic community connectedness (i.e. stigmatising experiences with non-autistic people, and pushed autistic people towards each other). This is a hypothesis generated from the present research that should be tested in future studies.

The accounts given by participants of autistic community connectedness demonstrate the five elements sense of community: membership, influence, reinforcement, shared emotional connection and conscious identification (McMillan, 1996; Obst et al., 2002). Participants described strong belongingness to the autistic community (McMillan, 1996). This relatedness was something many participants did not tend to feel elsewhere. Participants described influence, mattering and the ability to make a change, with a focus on what ‘I’ can do for the group. The data show reinforcement, both integration and needs fulfilment (McMillan, 1996). Finally, participants detailed a shared emotional connection, and most had a conscious identification with the autistic community. Furthermore, where other research (Lam et al., 2020) has explored autistic narratives which described a general connectedness to geographical community and the environments around them, this work specifically refers to a psychological connectedness and feeling of community between autistic people, thus broadening the understanding of sense of community experienced by autistic people.

Belongingness to the community has been described elsewhere in the same ‘instantaneous’ manner (Bagatell, 2010), as in these data. This feeling of ‘home’ could relate to the sense of belongingness referred to by Baumeister and Leary (1995). In participants’ accounts, a sense of belongingness allowed participants to develop a sense of self-worth. Researchers have found that sense of belonging predicts higher meaningfulness in life beyond social support or social value (Lambert et al., 2013). Furthermore, for minority communities, belongingness to the in-group community is associated with increased wellbeing (Barr et al., 2016; Kaniuka et al., 2019) and the role of belongingness in relation to wellbeing should be considered in the future for the autistic community.

In social connectedness, accessibility was key, whereby autistic made spaces that were more welcoming. This is also supported in wider literature about what an ‘autistic space’ is: Buckle (2020) describes ‘events for autistic people that are organised by neurotypicals can be autistic-friendly, but they will never be truly autistic spaces’ (p. 118). Participants described how going to autistic-led events meant a degree of certainty that space will be for them. Architecture and space are not designed with autistic individuals in mind (Toronyi, 2019). Participants described a relatedness with environments that suited their needs and allowed them to cultivate relationships.

Political connectedness was a key component of autistic community connectedness. Participants described how their political role was to advocate against stigma, violence against autistic people and ‘snake-oil’ cures, such as the supposed Miracle Mineral Supplement [MMS; bleach] treatments. The goals to end stigmatising campaigns, educate the public, progress bans on unethical cures and direct research funding into areas considered important to those of the community (Dalamayne, 2017; Kapp, 2020; Kras, 2010; Lewis, 2016; Pellicano et al., 2014).

Participants described refusing to take part in genetic research which may lead to the potential for the removal of autistic genes or a cure. Prior commentary has found that autistic people often feel detached and dehumanised by autism research (Cowen, 2009; Gernsbacher, 2007; Luterman, 2019; Rose, 2020). It is unsurprising that autistic people may inadvertently control the direction of autism research by refusing to engage with studies they feel are unethical. This finding may also represent the frustration that the funding landscape in autism research is far removed from the needs and wants of autistic people (Pellicano et al., 2014b).

The benefits of political connectedness included connecting to other autistic people who shared their goals and having a sense of direction by aligning with neurodiversity. This goal was specifically to better the place of minorities in society, which is not unique to the data presented in this study (Bagatell, 2010; Kapp, 2020). Furthermore, it appeared that political connectedness allowed people a chance to challenge stigmatising representations and narratives. Challenging stigma may have important implications for ensuring it does not become ‘attached to the self’ and degenerate into internalised stigma (Wang et al., 2017).

Political connectedness was described as connectedness for a reason. It is not simply a motivation to further the needs and rights of autistic individuals; instead it is akin to being connected to a ‘power grid’ of disability tradition of reclaiming human dignity (Tisoncik, 2020), whereby other autistic individuals are ‘comrades’. It is not motivation because motivation is individualistic, and the political movements in autism were born out of collectivism (Kapp, 2020). This political connectedness might further be understood through the lens of politicised collective identities (Simon & Klandermans, 2001) where autistic people have an awareness of shared grievances (genetic research and a fear of eradication), identify adversaries with whom they have a power-struggle with (researchers and professionals who advocate for these genetic understandings) and use neurodiversity to challenge this power and gain support from a third party (the general public) for their understanding of autism.

This connectedness may also be conceptualised within what is termed ‘Imagined Communities’ (Anderson, 1991): members of the autistic community cannot possibly know all autistic people face-to-face as individuals, but may identify broadly as a collective and share a real affinity with one and other regardless, almost as a nation of people sharing a psychological space. Participants’ narratives clearly reflected the space and solidarity that autistic people made for each other, and experienced with each other, akin to the observations of Ryan Idriss from observing a North American autistic-led group (Ryan Idriss, 2021). Ryan Idriss makes a pertinent point that ‘autistic and other disability communities, are at work building invisible infrastructures in communities across the globe’ (2020; p. 9), and this was clear given that participants actively aimed to create and partake in spaces where autistic people could be themselves, experience belonging, socialise on their own terms and partake in creating enduring political change for the autistic people who come next.

A few participants experienced disconnectedness from the autistic community, supporting the necessity of conscious identification (Obst et al., 2002). Some of these individuals displayed what could be argued as internalised stigma (whereby they acknowledge that they are autistic, but stress that they are not like *other* autistic individuals). These same participants described an ambivalent relationship to an autism identity. Interestingly, this is one of the ways that identity acted as a gatekeeper to autistic community connectedness – participants who did not have a strong identity, or those who experienced self-stigma were disconnected from communities (autistic and neurotypical). This is like Bagatell’s (2010) case study of the teenager discovering the autistic community, but feeling an increased sense of stigma, and that identification with the community would bring him no closer to connection with the non-autistic community.

However, that is not to say that all autistic people who are not connected to the community may have internalised stigma, nor that connectedness is a completely ideal state – the autistic community also has systemic issues as other communities do, which may make it harder for multiply marginalised people to engage. Racism is a systemic problem throughout autism rhetoric (Czech, 2018; Heilker, 2012), in research (Jones & Mandell, 2020; Jones et al., 2020), services and in the diagnosis process (Mandell et al., 2009; Slade, 2014). Nuance is needed when considering the reasons why people may or may not experience autistic community connectedness.

These findings have important implications for both how different autistic people might deal with the exposure to minority stress, and also for understanding wellbeing in autistic people (Botha & Frost, 2020). Increased group identification can buffer against the effect of discrimination (Major et al., 2003) but only for those who hold that identity central (Major & O’Brien, 2005). This suggests that autistic community connectedness may buffer against the impact of minority but perhaps only for those who hold the identification closely. Our findings also qualitatively supports findings that for some individuals who have internalised stigma, increased belongingness may be associated with increased self-stigma (Lambert et al., 2013). Belongingness may be able to reduce stigma and discrimination, but not the internalisation of it, making internalised stigma particularly insidious. While belongingness or increased group identification may not help with this, political connectedness may play a role as it involves challenging these narratives specifically. Overall, most participants expressed the role of autistic community connectedness for bringing joy, companionship, and as such further consideration for autistic community connectedness as a source of wellbeing should be explored in future research.

Some participants were not emotionally or socially connected to the autistic community but still engaged in collective action. This may mean different types of autistic community connectedness result in individual versus collective engagement. In our related work on stigma and identity (Botha et al., 2020), participants described a process of language reclamation and strategic disclosure to unsettle stigmatising narratives around autism. Furthermore, challenging negative narratives around autism requires challenging one’s own belief in them. It is important to consider how identity, political connectedness and aspects such as language reclamation and reframing might work together to buffer against exposure to minority stressors and how this relates to a politicised collective identity (Simon & Klandermans, 2001) and collective action (Velez & Moradi, 2016).

### Reflection

Five tentative criteria for establishing transgressive validity have been suggested for qualitative research (Dennis, 2013; Lather, 2009:): it should be substantive and add to understanding of social life; have ‘aesthetic merit’; address the complexity of representation through reflexivity; produce positive impact for participants; and be experience near (Dennis, 2013; p. 7). This body of work aims to strengthen and deepen an understanding of the social and cultural life of autistic people, and to add to what has been elsewhere described as an ‘autistic sociality’ (Ryan Idriss, 2021). The rich data transgress the predominant biomedical understandings of autism which construct autism as a social communicational disorder with inherent impairments of theory of mind, which elsewhere, have been said to make an autistic community ‘impossible’ (Barnbaum, 2008). This work also includes narratives from participants who are non-speaking and often denied sociality or agency in autism literature. Their inclusion contributes to the studies aesthetic merit by providing vibrant accounts of autistic sociality from those who are often unjustly ignored. This study has a limited ability to make a difference for the participants beyond having their voices raised on to a platform but may make a bigger difference for autistic people more generally by challenging the biomedical notion of autistic social deficits.

More complicated, is whether the study addresses the complexities of representation through reflexive and participatory engagement. The use of critical realist grounded theory demanded a reflexive practice because critical realism highlights that, despite a singular reality, all representations of it through knowledge are historically, culturally and socially situated (Oliver, 2012). Our focus was on creating ethical a rights-based, ethical, and affirmative research process and product. To do this, we coded all the data to reduce how much power we had as researchers to be selective. Furthermore, we used the constant comparison approach to continually adapt to incoming data, alter our questions to reflect the direction of participants’ narratives, and collected data using multiple methods of data collection to be inclusive. This iterative process was central to the use of grounded theory tools and means that we were nearer to experience than we would have been if we had used non-reflexive, non-adaptive, rigid methods. Despite this, a fully participatory approach would have been beneficial. While the first author is autistic, it is not their duty nor place to speak for the wider autistic community and participatory research can embody more autistic voice into autism research. This approach is particularly important given that autistic individuals at other intersections of identity (like Black autistic individuals) are under-represented (Jones & Mandell, 2020).

### Limitations and future research

Although our methods of data collection were varied, allowing us to interview participants who do not communicate in conventional ways, we did not interview anyone who disclosed a co-occurring learning disability. Future research should address autistic community connectedness for people with co-occurring learning disabilities who might have increased barriers for accessing community. Furthermore, the role and benefits of autistic community connectedness in buffering against stigma and minority stress would be important to explore given how prevalent stigma (Botha et al., 2020; Butler & Gillis, 2011; Holton et al., 2014) and minority stress (Botha & Frost, 2020) are in autistic people’s lives. Research should, however, also focus on individuals who are disconnected as these people might be more vulnerable to increased exclusion – a limitation of this article is that it particularly focused on connectedness. Future work should explore the nuance of being disconnected from the autistic community. Finally, given that the sample was predominantly diagnosed autistic people, future work should aim to recruit a balanced sample, or a sample of self-diagnosed autistic people to understand if there are nuanced differences between these groups with regards to autistic community connectedness, given that while self-diagnosed people also experience a belongingness to other autistic people, they might experience diagnostic doubt or struggle with identity (Lewis, 2016).

## Conclusion

The aim of the study was to investigate autistic community connectedness using grounded theory tools to centre the experiences and narratives of autistic people. Although preliminary, these results establish a grounded, experienced-based construct of autistic community connectedness. The multifaceted community which includes elements of belongingness, social connectedness and political connectedness is a vibrant, and welcoming space for autistic people – having said that, not all autistic participants experienced connectedness to the community. More research is needed to expand our knowledge of autistic community connectedness, and the ways in which autistic people experience community with one and other, and its resulting implications for autistic people’s wellbeing. Attention in future research should be paid to autistic community connectedness, including potential benefits of connectedness, along with facilitators and barriers to experiencing connectedness to a community of autistic people.

## Supplemental Material

sj-docx-1-aut-10.1177_13623613221080248 – Supplemental material for ‘It’s being a part of a grand tradition, a grand counter-culture which involves communities’: A qualitative investigation of autistic community connectedness Click here for additional data file. Supplemental material, sj-docx-1-aut-10.1177_13623613221080248 for ‘It’s being a part of a grand tradition, a grand counter-culture which involves communities’: A qualitative investigation of autistic community connectedness by Monique Botha, Bridget Dibb and David M Frost in Autism

## Notes

1. Although this reflects the language of Steve Silberman’s book NeuroTribes, and was used by multiple participants, there are issues of appropriation of culture and language that need to be considered in continuing the use of this term here, including continuing to reproduce its use by white autistic people uncritically.

## References

[bibr1-13623613221080248] Åge L.-J. (2011). Grounded theory methodology: Positivism, hermeneutics, and pragmatism (Vol. 16). https://nsuworks.nova.edu/tqr/vol16/iss6/8/#:~:text=Glaserian%20grounded%20theory%20methodology%2C%20which,comprehensive%20analysis%20of%3A%20(a)

[bibr2-13623613221080248] Allen K.-A. (2021). The psychology of belonging. Routledge, Taylor & Francis Group. https://www.taylorfrancis.com/books/9780429327681

[bibr3-13623613221080248] Anderson B. R. O. (1991). Imagined communities: Reflections on the origin and spread of nationalism (Rev. and extended ed.). Verso Books.

[bibr4-13623613221080248] Bagatell N. (2007). Orchestrating voices: Autism, identity and the power of discourse. Disability & Society, 22(4), 413–426. 10.1080/09687590701337967

[bibr5-13623613221080248] Bagatell N. (2010). From cure to community: Transforming notions of autism. Ethos, 38(1), 33–55. 10.1111/j.1548-1352.2009.01080.x

[bibr6-13623613221080248] Barad K. M. (2007). Meeting the universe halfway: Quantum physics and the entanglement of matter and meaning. Duke University Press.

[bibr7-13623613221080248] Barnbaum D. R. (2008). The ethics of autism: Among them, but not of them. Indiana University Press. http://public.eblib.com/choice/publicfullrecord.aspx?p=415343

[bibr8-13623613221080248] Barr S. M. Budge S. L. Adelson J. L. (2016). Transgender community belongingness as a mediator between strength of transgender identity and well-being. Journal of Counseling Psychology, 63(1), 87–97. 10.1037/cou0000127 26751157

[bibr9-13623613221080248] Baumeister R. F. Leary M. R. (1995). The need to belong: Desire for interpersonal attachments as a fundamental human motivation. Psychological Bulletin, 117(3), 497–529. 7777651

[bibr10-13623613221080248] Bauminger N. Solomon M. Aviezer A. Heung K. Brown J. Rogers S. J. (2008). Friendship in high-functioning children with autism spectrum disorder: Mixed and non-mixed dyads. Journal of Autism and Developmental Disorders, 38(7), 1211–1229. 10.1007/s10803-007-0501-2 18058212 PMC5538879

[bibr11-13623613221080248] Bhaskar R. (1997). A realist theory of science. Verso.

[bibr12-13623613221080248] Botha M. Dibb B. Frost D. (2020). ‘Autism is me’: An investigation of how autistic individuals make sense of autism and stigma. Disability and Society. Advance online publication. 10.1080/09687599.2020.1822782

[bibr13-13623613221080248] Botha M. Frost D. M. (2020). Extending the minority stress model to understand mental health problems experienced by the autistic population. Society and Mental Health, 10(1), 20–34. 10.1177/2156869318804297

[bibr14-13623613221080248] Bronfenbrenner U. (1996). The ecology of human development: Experiments by nature and design. Harvard University Press.

[bibr15-13623613221080248] Buckle K. L. (2020). Autscape. In Kapp S. K. (Ed.), Autistic community and the neurodiversity movement: Stories from the frontline (pp. 109–122). Springer.

[bibr16-13623613221080248] Butler R. C. Gillis J. M. (2011). The impact of labels and behaviors on the stigmatization of adults with Asperger’s disorder. Journal of Autism and Developmental Disorders, 41(6), 741–749. 10.1007/s10803-010-1093-9 20811769

[bibr17-13623613221080248] Cassidy S. Bradley P. Robinson J. Allison C. McHugh M. Baron-Cohen S. (2014). Suicidal ideation and suicide plans or attempts in adults with Asperger’s syndrome attending a specialist diagnostic clinic: A clinical cohort study. The Lancet Psychiatry, 1(2), 142–147. 10.1016/S2215-0366(14)70248-2 26360578

[bibr18-13623613221080248] Charmaz . (2008). Constructionism and the grounded theory method. In Holstein J. A. Gumbrium J. F. (Eds.), Handbook of constructionist research (pp. 397–412). Guildford Press.

[bibr19-13623613221080248] Charmaz K. (2006). Constructing grounded theory: A practical guide through qualitative analysis. SAGE.

[bibr20-13623613221080248] Chavis D. M. Hogge J. H. McMillan D. W. Wandersman A. (1986). Sense of community through Brunswik’s lens: A first look. Journal of Community Psychology, 14(1), 24–40. 10.1002/1520-6629(198601)14:1<24::AID-JCOP2290140104>3.0.CO;2-P

[bibr21-13623613221080248] Cooper K. Smith L. G. E. Russell A. (2017). Social identity, self-esteem, and mental health in autism: Social identity, self-esteem, and mental health in autism. European Journal of Social Psychology, 47(7), 844–854. 10.1002/ejsp.2297

[bibr22-13623613221080248] Corbin J. Strauss A. L. (2008). Basics of qualitative research: Techniques and procedures for developing grounded theory (3rd ed.). SAGE. 10.4135/9781452230153

[bibr23-13623613221080248] Cowen T. (2009). Autism as academic paradigm. The Chronicles of Higher Education. http://www.chronicle.com/article/Autism-as-Academic-Paradigm/47033

[bibr24-13623613221080248] Crocker J. Major B. (1989). Social stigma and self-esteem: The self-protective properties of stigma. Psychological Review 96(4): 608–630. 10.1037/0033-295x.96.4.608

[bibr25-13623613221080248] Crompton C. J. Fletcher-Watson S. Ropar D. (2019). “I never realised everybody felt as happy as I do when I am around autistic people”: A thematic analysis of autistic adults’ relationships with autistic and neurotypical friends and family. [Preprint]. Open Science Framework. 10.31219/osf.io/46b87 PMC7376620 32148068

[bibr26-13623613221080248] Crompton C. J. Ropar D. Evans-Williams C. V. Flynn E. G. Fletcher-Watson S. (2020). Autistic peer-to-peer information transfer is highly effective. Autism, 24(7), 1704–1712. 10.1177/1362361320919286 32431157 PMC7545656

[bibr27-13623613221080248] Crompton C. J. Sharp M. Axbey H. Fletcher-Watson S. Flynn E. G. Ropar D. (2020). Neurotype-matching, but not being autistic, influences self and observer ratings of interpersonal rapport. Frontiers in Psychology, 11, Article 586171. 10.3389/fpsyg.2020.586171 PMC7645034 33192918

[bibr28-13623613221080248] Czech H. (2018). Hans Asperger, national socialism, and ‘race hygiene’ in Nazi-era Vienna. Molecular Autism, 9(1), 1–43. 10.1186/s13229-018-0208-6 29713442 PMC5907291

[bibr29-13623613221080248] Dalamayne E. (2017). Make autistic “cures” illegal in the UK. https://www.change.org/p/phillip-dunne-make-autistic-cures-illegal-in-the-uk

[bibr30-13623613221080248] Deaux K. (1996). Social identification. In Van Lange P. A. M. Tory Higgins E. Kruglanski A. W. Aknin L. B. Algoe S. B. (Eds.), Social psychology: Handbook of basic principles (pp. 777–798). Guilford Press.

[bibr31-13623613221080248] de Hooge A . (2019). Binary boys: Autism, Aspie supremacy and post/humanist normativity. Disability Studies Quarterly, 39, Article 6461. 10.18061/dsq.v39i1.6461

[bibr32-13623613221080248] Dennis B. (2013). ‘Validity crisis’ in qualitative research. In Carspecken H. L. Carspecken P. F. Dennis B. (Eds.), Qualitative research: A reader in philosophy, core concepts, and practice (pp. 3–37). Peter Lang.

[bibr33-13623613221080248] Douglas H. (2010). Types of community. In Anheier H. K. Toepler S. (Eds.), International encyclopedia of civil society (pp. 539–544). Springer. 10.1007/978-0-387-93996-4_542

[bibr34-13623613221080248] Frost D. Meyer I. (2012). Measuring community connectedness among diverse sexual minority populations. Journal of Sex Research, 49(1), 36–49. 10.1080/00224499.2011.565427 21512945 PMC3143245

[bibr35-13623613221080248] Gernsbacher M. A. (2007). On not being human. APS Observer, 20(2), 5–32. PMC4266404 25520547

[bibr36-13623613221080248] Glaser B. G. Strauss A. L. (1967). The discovery of grounded theory: Strategies for qualitative research. Aldine.

[bibr37-13623613221080248] Hadley G. (2019). Critical grounded theory. In Bryant A. Charmaz K. (Eds.), The Sage handbook of current developments in grounded theory (pp. 564–592). SAGE. 10.4135/9781526485656.n30

[bibr38-13623613221080248] Hannon G. Taylor E. P. (2013). Suicidal behaviour in adolescents and young adults with ASD: Findings from a systematic review. Clinical Psychology Review, 33(8), 1197–1204. 10.1016/j.cpr.2013.10.003 24201088

[bibr39-13623613221080248] Heilker P. (2012). Autism, rhetoric, and whiteness. In Falkof N. Cashman-Brown O. (Eds.), On whiteness (pp. 193–204). Brill. 10.1163/9781848881051_020

[bibr40-13623613221080248] Hoddy E. T. (2018). Critical realism in empirical research: Employing techniques from grounded theory methodology. International Journal of Social Research Methodology, 22(1), 111–124. 10.1080/13645579.2018.1503400

[bibr41-13623613221080248] Holton A. E. Farrell L. C. Fudge J. L. (2014). A threatening space? Stigmatization and the framing of autism in the news. Communication Studies, 65(2), 189–207. 10.1080/10510974.2013.855642

[bibr42-13623613221080248] Janesick V. J. (2015). Journaling, Reflexive. In Ritzer G. (Ed.), The blackwell encyclopedia of sociology (p. wbeosj007.pub2). John Wiley & Sons, Ltd.

[bibr43-13623613221080248] Jones D. R. Mandell D. S. (2020). To address racial disparities in autism research, we must think globally, act locally. Autism, 24(7), 1587–1589. 10.1177/1362361320948313 32998555

[bibr44-13623613221080248] Jones D. R. Nicolaidis C. Ellwood L. J. Garcia A. Johnson K. R. Lopez K. Waisman T. (2020). An expert discussion on structural racism in autism research and practice. Autism in Adulthood, 2(4), 273–281. 10.1089/aut.2020.29015.drj PMC8992862 36600959

[bibr45-13623613221080248] Kaniuka A. Pugh K. C. Jordan M. Brooks B. Dodd J. Mann A. K. Williams S. L. Hirsch J. K. (2019). Stigma and suicide risk among the LGBTQ population: Are anxiety and depression to blame and can connectedness to the LGBTQ community help? Journal of Gay & Lesbian Mental Health, 23(2), 205–220. 10.1080/19359705.2018.1560385

[bibr46-13623613221080248] Kapp S. K. (2020). Autistic community and the neurodiversity movement: Stories from the frontline. 10.1007/978-981-13-8437-0

[bibr47-13623613221080248] Kempster S. Parry K. W. (2011). Grounded theory and leadership research: A critical realist perspective. The Leadership Quarterly, 22(1), 106–120. 10.1016/j.leaqua.2010.12.010

[bibr48-13623613221080248] Kempster S. Parry K. W. (2014). Critical realism and grounded theory. In Edwards P. K. O’Mahoney J. Vincent S. (Eds.), Studying organizations using critical realism: A practical guide. Oxford University Press. https://oxford.universitypressscholarship.com/view/10.1093/acprof:oso/9780199665525.001.0001/acprof-9780199665525-chapter-5

[bibr49-13623613221080248] Kolb S. M. (2012). Grounded theory and the constant comparative method: Valid research strategies for educators. Journal of Emerging Trends in Educational Research and Policy Studies, 3(1), 83–86.

[bibr50-13623613221080248] Kras J. (2010). The “Ransom Notes” affair: When the neurodiversity movement came of age. Disability Studies Quarterly, 30(3).

[bibr51-13623613221080248] Lai M.-C. Kassee C. Besney R. Bonato S. Hull L. Mandy W. Szatmari P. Ameis S. H. (2019). Prevalence of co-occurring mental health diagnoses in the autism population: A systematic review and meta-analysis. The Lancet Psychiatry, 6(10), 819–829. 10.1016/S2215-0366(19)30289-5 31447415

[bibr52-13623613221080248] Lam G. Y. H. Holden E. Fitzpatrick M. Raffaele Mendez L. Berkman K. (2020). ‘Different but connected’: Participatory action research using photovoice to explore well-being in autistic young adults. Autism, 24(5), 1246–1259. 10.1177/1362361319898961 31968999

[bibr53-13623613221080248] Lambert N. M. Stillman T. F. Hicks J. A. Kamble S. Baumeister R. F. Fincham F. D. (2013). To belong is to matter: Sense of belonging enhances meaning in life. Personality and Social Psychology Bulletin, 39(11), 1418–1427. 10.1177/0146167213499186 23950557

[bibr54-13623613221080248] Lather P. (2009). Getting lost: Feminist efforts toward a double(d) science. Frontiers: A Journal of Women Studies, 30(1), 222–230.

[bibr55-13623613221080248] Lewis L. F. (2016). Exploring the experience of self-diagnosis of autism spectrum disorder in adults. Archives of Psychiatric Nursing, 30(5), 575–580. 10.1016/j.apnu.2016.03.009 27654240

[bibr56-13623613221080248] Luterman. (2019). What it’s like to be autistic at an autism research conference. https://www.spectrumnews.org/opinion/what-its-like-to-be-autistic-at-an-autism-research-conference/

[bibr57-13623613221080248] Major B. O’Brien L. T. (2005). The social psychology of stigma. Annual Review of Psychology, 56(1), 393–421. 10.1146/annurev.psych.56.091103.070137 15709941

[bibr58-13623613221080248] Major B. Quinton W. J. Schmader T. (2003). Attributions to discrimination and self-esteem: Impact of group identification and situational ambiguity. Journal of Experimental Social Psychology, 39(3), 220–231. 10.1016/S0022-1031(02)00547-4

[bibr59-13623613221080248] Mandell D. S. Wiggins L. D. Carpenter L. A. Daniels J. DiGuiseppi C. Durkin M. S. Giarelli E. Morrier M. J. Nicholas J. S. Pinto-Martin J. A. Shattuck P. T. Thomas K. C. Yeargin-Allsopp M. Kirby R. S. (2009). Racial/ethnic disparities in the identification of children with autism spectrum disorders. American Journal of Public Health, 99(3), 493–498. 10.2105/AJPH.2007.131243 19106426 PMC2661453

[bibr60-13623613221080248] McMillan D. W. (1996). Sense of community. Journal of Community Psychology, 24(4), 315–325. 10.1002/(SICI)1520-6629(199610)24:4<315::AID-JCOP2>3.0.CO;2-T 32652592

[bibr61-13623613221080248] Meyer I. H. (2003). Prejudice, social stress, and mental health in lesbian, gay, and bisexual populations: Conceptual issues and research evidence. Psychological Bulletin, 129(5), 674–697. 10.1037/0033-2909.129.5.674 12956539 PMC2072932

[bibr62-13623613221080248] Meyer I. H. (2015). Resilience in the study of minority stress and health of sexual and gender minorities. Psychology of Sexual Orientation and Gender Diversity, 2(3), 209–213. 10.1037/sgd0000132

[bibr63-13623613221080248] Obst P. Zinkiewicz L. Smith S. G. (2002). Sense of community in science fiction fandom, Part 1: Understanding sense of community in an international community of interest. Journal of Community Psychology, 30(1), 87–103. 10.1002/jcop.1052

[bibr64-13623613221080248] Oliver C. (2012). Critical realist grounded theory: A new approach for social work research. British Journal of Social Work, 42(2), 371–387.

[bibr65-13623613221080248] Pellicano E. Dinsmore A. Charman T. (2014). What should autism research focus upon? Community views and priorities from the United Kingdom. Autism, 18(7), 756–770. 10.1177/1362361314529627 24789871 PMC4230972

[bibr66-13623613221080248] Pring J. (2019). Labour conference: McDonnell says autistic activists must ‘eyeball’ shadow ministers. Disability News Services. https://www.disabilitynewsservice.com/labour-conference-mcdonnell-says-autistic-activists-must-eyeball-shadow-ministers/

[bibr67-13623613221080248] QSR International Pty Ltd. (2015). NVivo (version 11) https://www.qsrinternational.com/nvivo-qualitative-data-analysis-software/home

[bibr68-13623613221080248] Rose K. (2020). Regarding the use of dehumanising rhetoric. https://theautisticadvocate.com/2020/02/regarding-the-use-of-dehumanising-rhetoric/

[bibr69-13623613221080248] Ryan Idriss C . (2021). Invisible autistic infrastructure: Ethnographic reflections on an autistic community. Medical Anthropology, 40(2), 129–140. 10.1080/01459740.2020.1849185 33216640

[bibr70-13623613221080248] Silberman S. (2015). Neurotribes. Allen & Unwin.

[bibr71-13623613221080248] Simon B. Klandermans B. (2001). Politicized collective identity: A social psychological analysis. American Psychologist, 56(4), 319–331. 10.1037/0003-066X.56.4.319 11330229

[bibr72-13623613221080248] Sinclair. (2005). Autism network international: The development of a community and its culture. https://web.archive.org/web/20090126110607/http://web.syr.edu/~jisincla/History_of_ANI.html

[bibr73-13623613221080248] Slack R. C. (1998). What is a community? Public Health, 112(6), Article 361. 10.1038/sj.ph.1900514 9883029

[bibr74-13623613221080248] Slade G. (2014). Diverse perspectives: The challenges for families affected by autism from Black, Asian and Minority Ethnic communities. https://www.autism.org.uk/advice-and-guidance/what-is-autism/autism-and-bame-people

[bibr75-13623613221080248] Tisoncik L. A. (2020). Autistics.org and finding our voices as an activist movement. In Kapp S. K. (Ed.), Autistic community and the neurodiversity movement (pp. 65–76). Springer. 10.1007/978-981-13-8437-0_5

[bibr76-13623613221080248] Toronyi D. (2019). Hidden geographies: Design for neurodivergent ways of hearing and sensing. Cities & Health, 5, 133–137. 10.1080/23748834.2019.1627059

[bibr77-13623613221080248] Van Orden K. A. Witte T. K. Cukrowicz K. C. Braithwaite S. R. Selby E. A. Joiner T. E . (2010). The interpersonal theory of suicide. Psychological Review, 117(2), 575–600. 10.1037/a0018697 20438238 PMC3130348

[bibr78-13623613221080248] Velez B. L. Moradi B. (2016). A moderated mediation test of minority stress: The role of collective action. The Counseling Psychologist, 44(8), 1132–1157. 10.1177/0011000016665467

[bibr79-13623613221080248] Vollstedt M. Rezat S. (2019). An introduction to grounded theory with a special focus on axial coding and the coding paradigm. In Kaiser G. Presmeg N. (Eds.), Compendium for early career researchers in mathematics education (pp. 81–100). Springer. 10.1007/978-3-030-15636-7_4

[bibr80-13623613221080248] Wang C. S. Whitson J. A. Anicich E. M. Kray L. J. Galinsky A. D. (2017). Challenge your stigma: How to reframe and revalue negative stereotypes and slurs. Current Directions in Psychological Science, 26(1), 75–80. 10.1177/0963721416676578

[bibr81-13623613221080248] Ward M. J. Meyer R. N. (1999). Self-determination for people with developmental disabilities and autism: Two self-advocates’ perspectives. Focus on Autism and Other Developmental Disabilities, 14(3), 133–139. 10.1177/108835769901400302

[bibr82-13623613221080248] Wood C. Freeth M. (2016). Students’ stereotypes of autism. Journal of Educational Issues, 2(2), Article 131. 10.5296/jei.v2i2.9975

