# Supplementary material for: ‘It’s being a part of a grand tradition, a grand counter-culture which involves communities’: A qualitative investigation of autistic community connectedness
Source: Autism. 2022 Mar 23;26(8):2151–64. doi: 10.1177/13623613221080248 (PMC9597163; doi:10.1177/13623613221080248)
Supplement: sj-docx-1-aut-10.1177_13623613221080248 – Supplemental material for ‘It’s being a part of a grand tradition, a grand counter-culture which involves communities’: A qualitative investigation of autistic community connectedness [file sj-docx-1-aut-10.1177_13623613221080248.docx]

**Appendix A****: Interview schedule**

**Demographic information:**

The participant will be asked to provide certain demographic information:

1. Gender
2. Age
3. Diagnosis status (official or suspected and diagnosis type)
4. Nationality
5. Ethnicity
6. Sexual orientation
7. Relationship status
8. Educational status

**Guideline list of potential questions:**

-NOTE: Some of these questions may change over the course of the study, however the changes will be minor and the questions below demonstrate the overall thematic nature of the study:

**Questions on autism and diagnosis history**

1. Can you tell me about the age at which you were first diagnosed (or first suspected you were on the autism spectrum?
2. What was the process of being diagnosed like for you?
3. Did being diagnosed (or suspecting) you were on the autism spectrum, change the way you thought about yourself or your life?
   1. If yes, why/how/what changed?
   2. If not, why not?

**Questions on autism and identity?**

1. Do you feel like being on the autism spectrum is a core part of your identity (yes/no- why?)
2. Some people prefer to be described as on the autism spectrum, others as autistic. Do you have any preference and if so, can you explain why?
3. How often do you tell people who are not on the spectrum that you are? And what is their response? (If you do not, what is the reason?) Can you tell me about a time someone could tell? And how did it go when you have disclosed it?
4. How important is having a diagnosis to you? Do you feel it adds something to your identity or takes something away? (If you don’t have a diagnosis why so? Do you feel identifying with the community has added something to your life?
5. How do you feel when autism is discussed in the media? Has it ever changed the way you consider yourself or autism? For better or for worse?
6. When you experience being treated unfairly or negatively because of (being on the autism spectrum/being autistic), how do you cope with that experience?

**Questions on autism and community**

1. Do you have any other friends that are on the autism spectrum? (If so, what are these friendships like?)
2. In your experience have you found it easier or harder to connect to non-autistic individuals, compared to connecting to other people on the autism spectrum?
3. When autism is in the news for some reason of another, do you tend follow the story or its impact for the autism community? (If yes, what was the last big story you can remember that you followed and why did you follow it?)
4. When opportunities arise to take part in autism research, how often would you participate and why do you (or don’t you) participate? What does it mean to you?
5. Have there been any specific supports whether informal or formal that have made your life easier in some way?
6. Recently there have been a lot of communities developing on the internet for people on the spectrum. Are you apart of any of these online communities?
7. If yes to Q1, what was it that made you join?

If no to Q1, is there a reason you haven’t joined?

1. If you could choose now to go back and remain undiagnosed would you? Why?

Finishing questions:

1. Lastly, is there anything you would like to add or let me know?

**Questions added throughout the interviewing process**

1. How do you think society feels about autism?
2. How do you feel about autism?
3. Do you think there are stereotypes attached to autism? Why? Why not? What are they?
4. Do you feel you notice when someone else is also on the autism spectrum? If yes- how can you tell? What happens when you think someone else is? Does it attract you to them, or deter you from them?
5. Have you ever signed a petition relating to the autistic community? Why did you/ or not?
